# Supplementary material for: Comparative Analysis of the PIN Auxin Transporter Gene Family in Different Plant Species: A Focus on Structural and Expression Profiling of PINs in Solanum tuberosum
Source: Int J Mol Sci. 2019 Jul 3;20(13):3270. doi: 10.3390/ijms20133270 (PMC6650889; doi:10.3390/ijms20133270)
Supplement: Supplementary file 1 [file ijms-20-03270-s001.zip › Table S2.docx]

|  | Helix 1 | Helix2 | Helix 3 | Helix 4 | Helix 5 | Loop | Helix 6 | Helix 7 | Helix 8 | Helix 9 | Helix 10 |
| --- | --- | --- | --- | --- | --- | --- | --- | --- | --- | --- | --- |
| Refined model | 7L-28V | 39C-60S | 71F-93S | 100W-121L | 132L-152F |  | 5L-25F | 39C-60S | 71V-93A | 98C-120A | 130L-152L |
| StPIN1 | 7F-26G | 41G-60A | 67M-89W | 99E-121L | 128F-150F | 324 |  | 35I-57F | 70S-92I |  | 129I-151F |
| StPIN2 | 10V-29R | 42I-61T | 71F-93S | 100W-122K | 132L-154Y | 319 |  | 37G-59A | 74F-96L | 98G-120A | 130L-152L |
| StPIN3 |  |  |  |  |  | 310 | 5L-27W | 39I-61Q | 71V-93I | 98G-120A | 130L-152L |
| StPIN4 | 4W-26G | 39C-61M | 71F-93T | 100W-122I | 132L-154Y | 342 |  | 39I-61Q | 71V-93A | 98G-120A | 130L-152L |
| StPIN5 | 10V-32H | 45F-62I | 67L-89W | 96G-118V | 133V-155W | 41 |  | 37G-59A | 74Y-96L | 103I-120A | 130L-152L |
| StPIN6 | 4L-21F | 28G-46I | 56T-78I | 85W-107N | 117L-139Y | 231 | 13S-35V | 45A-67C | 74I-96L |  | 129I-151L |
| StPIN7 | 7L-26G | 41G-60A |  | 98L-120L | 130G-152F | 275 |  | 39I-61Q | 71I-93A | 98G-120A | 130L-152L |
| StPIN8 | 7V-26I |  | 71L-90A | 97N-119P | 132L-154L | 20 |  | 26A-48V |  |  |  |
| StPIN9 | 10V-28V | 41G-60A | 70R-89W | 96G-118I | 133M-152F | 282 |  | 39I-61S | 71I-93A | 98G-120A | 130L-152L |
| StPIN10 | 10V-32H | 45L-62V | 67M-89W | 96G-118I | 128V-150F | 35 | 8N-23V | 38C-60M | 73V-95G | 105I-127I |  |

**Table S2. The exact positions of the predicted helices within the trans-membrane domains in potato PIN proteins.**
