# Supplementary material for: Comparative Analysis of the PIN Auxin Transporter Gene Family in Different Plant Species: A Focus on Structural and Expression Profiling of PINs in Solanum tuberosum
Source: Int J Mol Sci. 2019 Jul 3;20(13):3270. doi: 10.3390/ijms20133270 (PMC6650889; doi:10.3390/ijms20133270)
Supplement: Supplementary file 1 [file ijms-20-03270-s001.zip › Table S4.docx]

**Table S4.** **Primers for qRT-PCR used in this study.**

| Primer Name | Sequence (in 5’→3’ order) |
| --- | --- |
| StPIN1-F | TTTGCTATGGCTGTGAGAT |
| StPIN1-R | TTGTGGTAGAGCTGCCTGT |
| StPIN2-F | TCATCTAAAGGGCCAACACC |
| StPIN2-R | GTTGTATAGCTCCCCGCTCA |
| StPIN3-F | TTGGTCCCTAATCTCGTAT |
| StPIN3-R | AAAGTAGCCACCGTGTTCC |
| StPIN4-F | ATGATTTTCTCGAAACAGACGC |
| StPIN4-R | CGGATTCCTCGAAGAACTAAGA |
| StPIN5-F | TGGCACATGTTTAAGCCAGA |
| StPIN5-R | GCCCATAAGACGAGGATCAG |
| StPIN6-F | GAATCCGCATTTTCATCCTC |
| StPIN6-R | CCCGTTATGTAAAGGCGTGT |
| StPIN7-F | CAGCCGAGCTGTTTCCTAAC |
| StPIN7-R | TTTTCGCCACACCATAATCA |
| StPIN8-F | ATCAGGAAGCAACCTCTACAAA |
| StPIN8-R | ACCAGACTAAACTCTGTAGTGC |
| StPIN9-F | GGCGAAAACTAATTAGGAACCC |
| StPIN9-R | GCTATAATCGCAGGCATTTGAA |
| StPIN10-F | GTGGTGACCTTATTGCTAAAGC |
| StPIN10-R | CAGGAATTCCCATAACAAGTGC |
