# Supplementary figures and images for: Comparative Analysis of the PIN Auxin Transporter Gene Family in Different Plant Species: A Focus on Structural and Expression Profiling of PINs in Solanum tuberosum
Source: Int J Mol Sci. 2019 Jul 3;20(13):3270. doi: 10.3390/ijms20133270 (PMC6650889; doi:10.3390/ijms20133270)

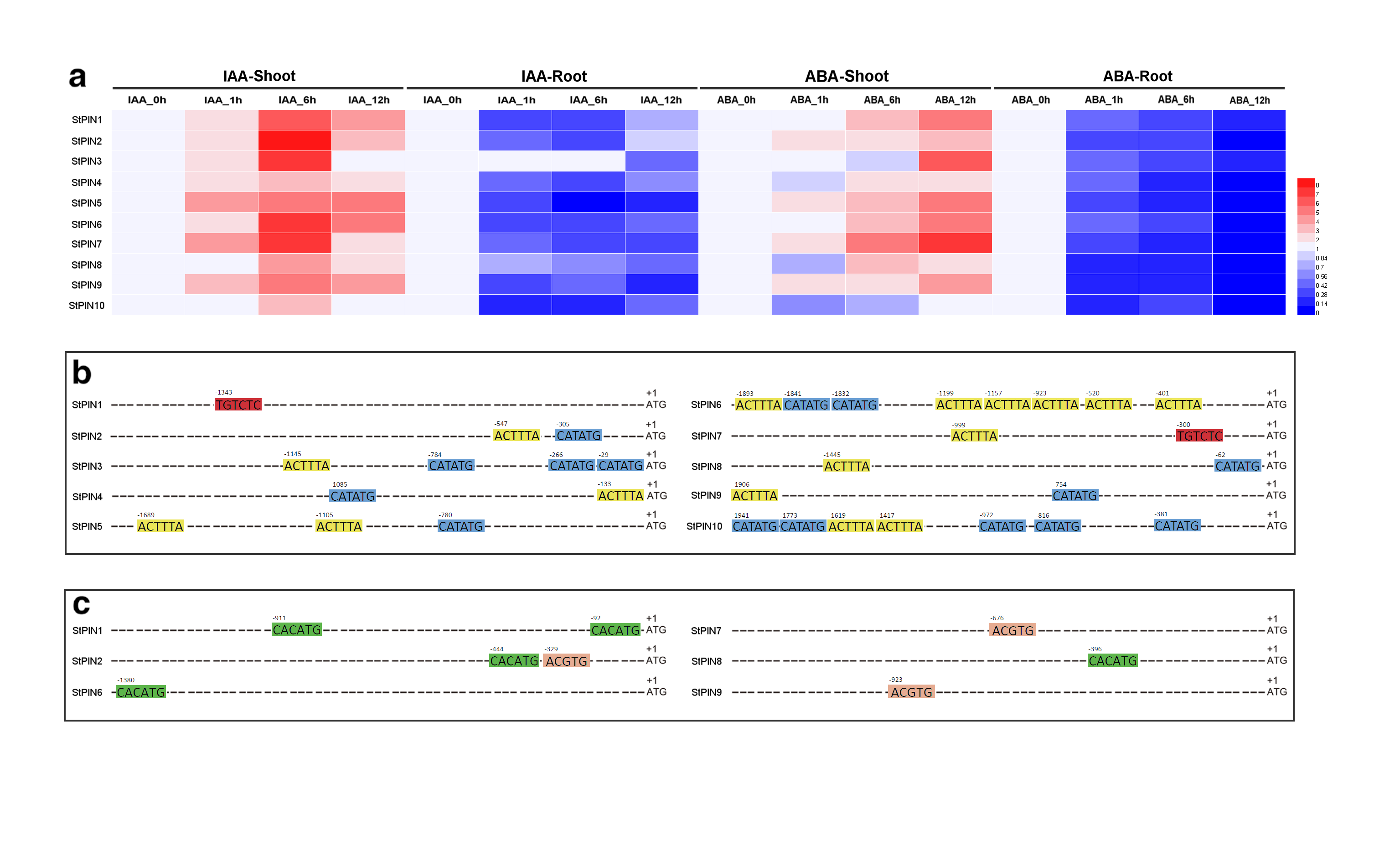

Supplement: Supplementary file 1 [file ijms-20-03270-s001.zip › Figure 5.tif]
